# Pediatric abdominal trauma in a National Referral Hospital

**DOI:** 10.4314/ahs.v22i2.16S

**Published:** 2022-08

**Authors:** Stella Alice Nimanya, John Sekabira, Nasser Kakembo, Phyllis Kisa, Alicia Massenga, Rovine Naluyimbazi, Felix Oyania, Innocent Okello

**Affiliations:** 1 Mulago National Referral Hospital. P.O.Box 7051, Kampala, Uganda; 2 Makerere University, College of Health Sciences. P.O.Box 7072, Kampala Uganda; 3 Mbarara University of Science and Technology. P.O. Box 1410, Mbarara, Uganda

**Keywords:** Pediatric trauma, abdominal injury

## Abstract

**Background:**

Trauma is a major contributor to pediatric morbidity and mortality. Injury and violence are a major killer of children throughout the world. Unintentional injuries account for almost 90% of these cases. They are the leading cause of death for children aged 10–19 years. More than 95% of all injury deaths in children occur in low income and middle-income countries. Abdominal trauma is present in approximately 25% of pediatric patients with major trauma and is the most common cause of unrecognized fatal injury in children.

**Objectives:**

To describe the patterns, the management and outcomes of pediatric abdominal trauma.

This was a descriptive retrospective study. Data was extracted from the Pediatric surgery Unit database from January 2012 to July 2019 on all abdominal trauma admissions to the unit.

**Results:**

Falls were the commonest (51.3%) mechanism for trauma on the unit. Most (84%) of the admissions had blunt abdominal trauma, with the majority (77%) managed non operatively. Only 16% had penetrating trauma, with the majority (84%) of these managed operatively. The average length of hospital stay for most (71.9%) of the patients was less than 7 days, with 96.1% of all admitted patients being discharged upon recovery.

**Conclusion:**

Blunt abdominal trauma is the most common pattern of pediatric abdominal trauma, with majority of these patients being managed non-operatively with good outcomes. Selective non-operative management for penetrating pediatric abdominal trauma has good patient outcomes as well.

## Introduction

### Background

Trauma causes significant pediatric morbidity and mortality. Worldwide, unintentional injuries are one of the top most causes of death and disability in the world affecting all populations, regardless of age, sex, income, or geographic region^1^. Initiatives to reduce child mortality worldwide have focused on reducing the burden of communicable diseases, however, the impact of non-communicable diseases on child health is being recognized 2.

According to a 2004 WHO report, injury and violence are a major killer of children throughout the world, responsible for about 950,000 deaths in children and young people under the age of 18 years each year 3. Unintentional injuries account for almost 90% of these cases. They are the leading cause of death for children aged 10–19 years 4. The most common causes of child injuries in Uganda are road traffic crashes, followed by falls and violent injuries^2^. More than 95% of all injury deaths in children occur in low income and middle-income countries, where in children over the age of 5 years, trauma causes more deaths than HIV, malaria, and tuberculosis combined^4^, ^5^. Although the child injury death rate is lower among children from developed countries, injuries are still a major cause of death, accounting for about 40% of all child deaths 3. Abdominal trauma is present in approximately 25% of pediatric patients with major trauma and is the most common cause of unrecognized fatal injury in children. Pediatric abdominal trauma is typically blunt in nature^6^.

Unpublished trauma related studies performed in our setting have indicated trauma as the single commonest indication for admission reported in the surgical units. Blunt abdominal injury is the commonest abdominal injury pattern seen at Mbarara Regional Referral in Uganda^7^.

Uganda is currently working towards transformation into middle income status by 2020. According to Uganda's most recent population census (2014), almost half (47.9%) of Uganda's population is aged between 0–14 years. With a growing increase in urbanization, also comes an increase in children's exposure to risk^8^. The total number of road traffic deaths and injuries from road traffic crashes is forecast to rise across the world by about 65% between 1990 and 2020 9, and in low income and middle income countries, deaths are expected to increase by as much as 80%. In Uganda, road traffic accidents are the most important cause of injury, causing 50% of all injuries^10^. However, this is different in the Ugandan pediatric population. Falls are the leading unintentional injury cause ahead of traffic among under age thirteen while burns lead below five years of age. Roads, homes and schools are lead locations of unintentional childhood injury with a male dominance^11^, ^12^.

A good portion of pediatric trauma patients in our setting usually end up being managed by General surgeons at health facilities of different levels all over the country as there no established pediatric trauma centres, no systematic pre-hospital care and not all referred patients make it to the Hospital that handles the largest proportion of trauma patients in the city centre. Patients are usually rushed to the nearest health facility following trauma. Trauma studies performed in our setting have mainly been adult based with small numbers of pediatric patients sampled and over short periods of time, thus missing a paucity of information in the pediatric population.

In a bid to reduce the morbidity and mortality associated with pediatric abdominal trauma, evidence-based protocols for management of children with abdominal injury are paramount, alongside child injury prevention strategies. Understanding the patterns of abdominal injury, and the outcomes following the current management pathways would aid greatly in developing protocols tailored to the pediatric population.

### Objectives

The objectives of the study were to describe the patterns, the management and the outcomes of pediatric abdominal trauma in a major trauma centre.

## Methods

This was a descriptive retrospective study at the Pediatric Surgical Unit.

The Paediatric surgical unit admits children aged 0–12 years only and has been enrolling all patients admitted to the unit since the year 2012 into a database that was approved by the Hospital Ethics board. This approval is renewed every year. Information is collected from patient charts retrospectively at discharge and includes patient demographics, admitting diagnosis, type of surgery performed if any and outcomes at discharge. However, investigations done during the admission, and findings at surgery are not entered into the database

All children under the age of 12 with a diagnosis of abdominal trauma were eligible for inclusion into the study. Children with burns, head trauma and extremity/bone injuries were not included in this study as they were admitted to their respective units, as opposed to the pediatric surgical unit.

A preformed data collection screen capturing the different study variables was designed using Microsoft excel. Data was collected from the pediatric surgery database from January 2012 to July 2019, on all patients admitted to the Pediatric Surgery unit during this time with abdominal trauma and entered into an excel spread sheet with the study variables and then analyzed using Stata version 14.

## Results

**Figure 1:**
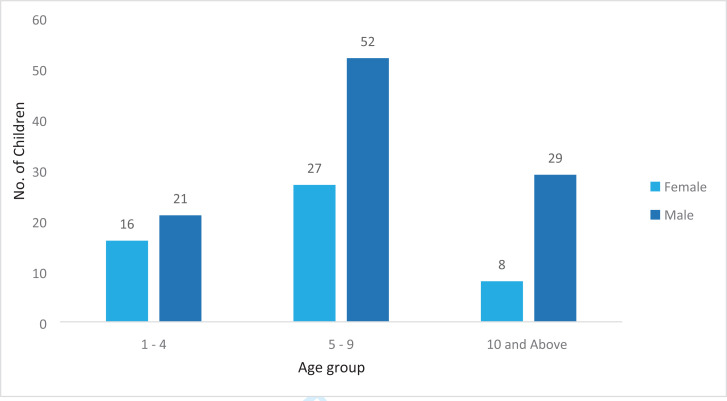
Patient demographics (sex and age ranges). Total number of patient records reviewed n=153.

A total of 153 patients had their records retrieved from the database. This is a small number for a period of 5 years as pediatric trauma patients are managed at various health facilities all over the country. Majority of the patients were male, aged between 5 and 9 years of age (Fig 1)

**Figure 2:**
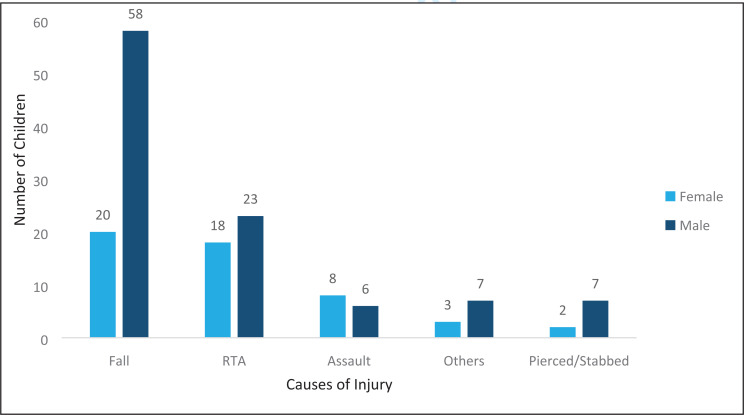
Distribution of patient sex in relation to the mechanisms of injury.

Falls accounted for more than half of the presenting injury mechanisms (Fig. 2)

**Figure 3:**
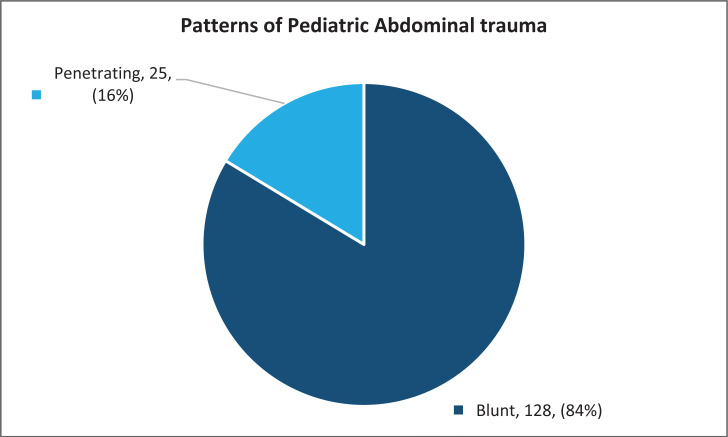
Patterns of Pediatric Abdominal trauma.

**Figure 4:**
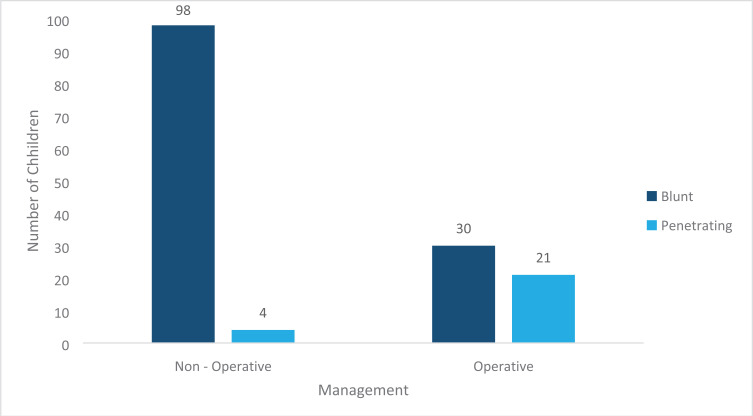
Management of the different patterns of Pediatric Abdominal Trauma.

Most (84%) of the patients admitted to the unit had blunt abdominal trauma, (Fig 3) and more than half (67) of all patients admitted were managed non-operatively (Fig. 4). In those presenting with penetrating trauma, the majority (84%) were managed operatively. Those managed non-operatively were selected based on their stable clinical status, with local wound exploration, repair of the defect and close monitoring.

**Figure 5:**
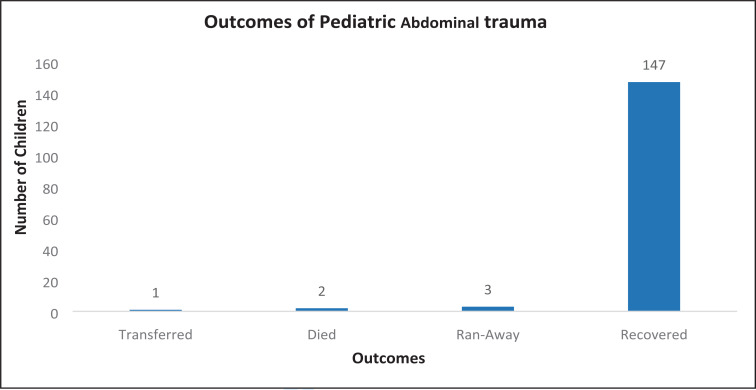
Outcomes of Pediatric Abdominal trauma

Most (96%) of the admitted patients recovered and were discharged (Fig 5). The average length of hospital stay was less than 7 days (71.9%).

## Discussion

Male children accounted for most (66.7%) of the patients admitted with abdominal trauma, which is similar to several other studies^13^–^15^. Children aged 5 to 9 years accounted for more than half of all admissions. This could be due to the fact that the unit admits patients aged 0–12yrs of age only, with the teenagers being managed on the adult service. Falls accounted for over half (51.3%) of all the injuries, and these were mainly falls from trees, majority being during the mango fruit season. This was consistent with other studies that have indicated falls as the main etiology of childhood trauma^11^, ^16^, ^17^. Majority (84%) of all admissions had blunt abdominal trauma, with the majority (77%) managed non operatively which is consistent with several other studies as to the pattern, however, many more patients with blunt abdominal trauma were managed operatively. This could be explained by the fact that some of these patients presented with features of peritonitis secondary to bowel perforation. There has been a notable increase in the number of bowel injuries following blunt abdominal trauma on the unit over the years. Unfortunately, the database does not include intraoperative findings and as such it is impossible to quantify and document the proportion of hollow viscus injuries. Selective non-operative management in penetrating abdominal trauma in pediatrics is controversial especially in a resource constrained setting in terms of adequate imaging, human resource and availability of emergency theatre services in the event of conversion to operative management. The average length of hospital stay for most (71.9%) of the patients was less than 7 days. This could be due to the fact that majority of the patients were managed non operatively with bed rest and monitoring until recovered. This could also be considered too long a stay especially for low grade (I and II) solid organ injuries. The database, however, does not classify the grades of injury for these trauma patients. A significant proportion (96.1%) of all admitted patients were discharged upon recovery. Only one patient was documented as transferred to anotherunit. Those with multiple organ injuries were more likely to be transferred to other units early on in the course of their management to enable multi-disciplinary management or managed by the pediatric surgical team while admitted on other units, hence this information wasn't captured in the pediatric surgical database, as their patient files would not have been available on the paediatric surgical unit.

## Conclusion

Blunt abdominal trauma is the most common pattern of pediatric abdominal trauma, with majority of these patients being managed non-operatively with good outcomes. Selective non-operative management for penetrating pediatric abdominal trauma has good patient outcomes as well.

We recommend using institution-based protocols in the management of pediatric abdominal trauma patients in a bid to optimize overall outcomes. Health sensitization campaigns should highlight the significant impact that falls from heights have on children, as the greatest mechanism for abdominal trauma. Prospective studies are recommended to further classify and describe the specific organs injured in these patients. Adjustments to the current pediatric surgery unit database at are also recommended to ensure it is comprehensive.
